# Interfacial Rheological Study of β-Casein/Pectin Mixtures at the Air/Water Interface

**DOI:** 10.3390/gels10010041

**Published:** 2024-01-03

**Authors:** Olga Mileti, Noemi Baldino, Stefania Luzzi, Francesca R. Lupi, Domenico Gabriele

**Affiliations:** Department of Information, Modeling, Electronics and System Engineering, (D.I.M.E.S.) University of Calabria, I-87036 Rende, Italy; o.mileti@dimes.unical.it (O.M.); stefania.luzzi@tno.nl (S.L.); francesca.lupi@unical.it (F.R.L.); d.gabriele@unical.it (D.G.)

**Keywords:** milk protein, biopolymers, surface tension

## Abstract

Colloidal food products, such as emulsions, foams, gels, and dispersions, are complex systems that need the presence of stabilizing agents to enable their formation and provide stability. Proteins are often used for food foams and emulsions because of their ability to lower interfacial tension and make viscoelastic interfaces. Generally, to improve the resistance against rupture, polysaccharides are used in association with the proteins. Pectin is a complex polysaccharide that can help to stabilize foams or emulsions. This work aims at studying the mechanical resistance of the interface formed by mixtures of β-casein and pectin at high and low methoxylation degrees at the air/water interface using dilatational and shear kinematics. Frequency sweep tests, in the linear region, were performed in shear at different aging times and in dilatational mode, and the rheological data were analyzed. The transient data of the surface tension were analyzed by kinetic models to obtain the characteristic rates of the interfacial phenomena. The kinetic mechanisms of the protein/pectin mixed systems are controlled by protein and show a weak gel behavior for short aging times. The interfaces obtained with both pectins in a mixture with β-casein evolved with time, gelling and showing a solid-like behavior at concentrations of 1 and 10 g/L and after 3.5 h of aging time. The interfacial shear trend obtained suggests a good stabilizing effect of the pectins from citrus with long aging times.

## 1. Introduction

Proteins and polysaccharides are biopolymers widely used in the food industry due to their ability to make and stabilize multiphasic systems such as foams, emulsions, gels, and dispersions [1,2]. Systems like foams or emulsions are characterized by the dispersion of one phase into another by mixing. The method used to obtain a foam or an emulsion has to be able to provide a large amount of energy to prevent them from evolving and giving rise to the phenomena of phase separation and instability within a short period of time [3]. Many of these systems can be created or stabilized by the addition of components that can rapidly absorb at the interface and make strong and structured viscoelastic layers [1,4]. Proteins are characterized by high interfacial activity and can form strong viscoelastic films [5,6], while polysaccharides are mainly used as thickening and gelling agents [7]. Some polysaccharides also have good interfacial activity and can interact with the species present at the interface, developing interactions that strongly stabilize the film [8,9]. The stabilization can be due to different phenomena, such as complexation, co-solubility, or segregation, resulting in synergistic or antagonistic effects [10,11,12]. The addition of proteins with polysaccharides creates a viscoelastic gel at the interface, and it also makes the gel resistant and with greater thickness [4]. For the design of new products, the interactions between proteins and polysaccharides have to be deeply understood.

Milk-based foods are a big slice of the food market, and the proteins from milk have been extensively investigated to create colloidal systems, emulsions, gels, and bioactive foods [12,13].

Milk proteins are subdivided into caseins and serum proteins; the former have random coil structures and the latter have a globular structure. Both, once absorbed at the interface, denature [14]. In particular, caseins make up 80% of the total proteins present in milk [15] and are characterized by four fractions: as1-casein, as2-casein, β-casein, and k-casein, which are present in a ratio of 4:1:4:1 [16,17]. They can be differentiated by their primary structures and molecular weights, which range between 19 and 25 kDa. The isoelectric point is between 4.1 and 5.3 [16]. β-casein forms about 30% of the casein proteins and has a principal hydrophobic behavior even if it is recognized, among the caseins, as the most soluble, highly amphiphilic, and calcium-sensitive phosphoprotein [13,18]. β-casein is rich in prolamine and poor in cysteine; it is not able to form intramolecular covalent disulfide bonds [15] and lacks a secondary structure because of the presence of uniformly distributed prolines in the complete amino acid sequence of β-casein [19,20]. Furthermore, the large amount of proline residues leads to the loss of secondary and tertiary structures [19,20]. This type of casein has high surface activity properties and is studied for applications in the food industry and for the stabilization of multiphasic systems [5,21]. Moreover, due to its unique amphiphilic structure, it can also be a natural nanocarrier for active ingredients [13,18].

In the last few years, the functional properties of colloidal systems have been improved thanks to the addition of other biopolymers like sodium alginate or xanthan gum [22]. In this regards, pectins have received particular attention in recent years, as already said, due to their health benefits. They are considered a safe food ingredient and a source of dietary fiber, as well as a good solution for lowering LDL cholesterol [23,24]. Pectins are complex polysaccharides that are obtained by acid extraction from the cell walls of plants [25]. They are used in the food industry as gelling agents, stabilizers, and texturizers [4,25]. In addition to the food industry, pectin is also used in other fields, such as cosmetics and pharmaceuticals [7]. Pectin is composed of monomeric units of D-galacturonic acid linked together with β-(1-4) bonds to form a linear backbone [26]. The pectin chains are constituted by homogalacturonan (HG), linear chains of partially methyl-esterified polygalacturonic acid, rhamnogalacturonan I (RG-I), and rhamnose residues [25,27]. Conventionally, the pectin structure is described as an HG backbone to which the other indicated structures are attached. By processes of methyl esterification and acetylation, methoxylated and acetylated units can be introduced. The ratio between esterified and acid groups in pectin molecules is defined as the degree of esterification (DE). Substituting a methyl for the OH group of the carboxylic acid is defined as methoxylation, and the percentage of methoxyl groups is defined as the degree of methoxylation (DM) [25,28].

According to the DM, it is possible to subdivide pectin into HM (pectin with a high degree of methoxylation (>50%)) and LM (pectin with a low degree of methoxylation (<50%)) [28,29]. Depending on their methoxylation degree, pectin exhibits different gelation mechanisms. LM pectin gels in the presence of bivalent cations (generally Ca^2+^) according to the egg-box model, which involves the linkage of galacturonan molecules through the formation of ionic and electrostatic bonds between groups [30]. HM pectin gels in acidic environments and with the presence of co-solutes [30]. Besides the recognized and investigated gelling and structuring properties [4,31,32], pectin shows good interfacial activity with regard to the chemical characteristics of the molecule, such as molecular weight, hydrophobicity, and protein residues present, but these depend on the source from which they are extracted [33,34,35]. In previous work, HM and LM pectins [24] showed good interfacial activity, with higher activity for LM than HM pectin, an interesting mechanical strength at the interface, and good structural and solid-like behavior at the air/water (A/W) interface, which are comparable with that of common protein species usually used for interfacial stabilization in food applications. In general, the presence of polysaccharides in a mixture with proteins can affect the protein adsorption and the behavior of the interfacial layer due to the development of several complex mechanisms of interaction between the molecules [36].

Nowadays, pectins are widely used in the food industry to stabilize dairy and dairy-based desserts. Different amounts of pectin can be added depending on the system to be designed. Generally, the ratio protein\pectin can range between 0.01 and 1, but in emulsions and aerated systems, a higher ratio can be used. In every case, the interfacial aspects are crucial for obtaining stable foods. Other literature works studied the mix of casein with both high-methoxyl (HM) and low-methoxyl (LM) pectins, but the interfacial aspects were not investigated [6,12]. Specifically, these studies found the pectin–casein system to be stable at a pH of about 6, and for the HM pectin–casein mixture, stability is observed throughout the pH range. Other literature studies have investigated the interfacial aspects of other milk proteins with pectins [22,37] as far as we know, but not β-casein.

In the pursuit of designing functional foods, the combination of β-casein/pectin could prove successful, given the protein’s carrying capacity and the health benefits of pectins [13]. Understanding the interfacial mechanisms associated with the utilization of proteins and polysaccharides plays a crucial role in formulating various colloidal systems. Therefore, due to the limited information on these systems, the present study aims to investigate the effect of competition and aggregation on the interfacial structure formation and stability of the β-casein/pectin systems. This serves as a model for developing novel bioactive food systems.

The potential competitive or cooperative behavior of pectins HM and LM in a mixture with β-casein, within the range where biopolymers can saturate the air–water interface on their own, was examined through measurements of surface pressure and surface dynamic properties in shear kinematics. Various aging times were considered, and dilatational kinematics of protein/pectin mixed systems at the A/W interface were performed.

## 2. Results and Discussion

### 2.1. Static Measurements

**Figure 1:**
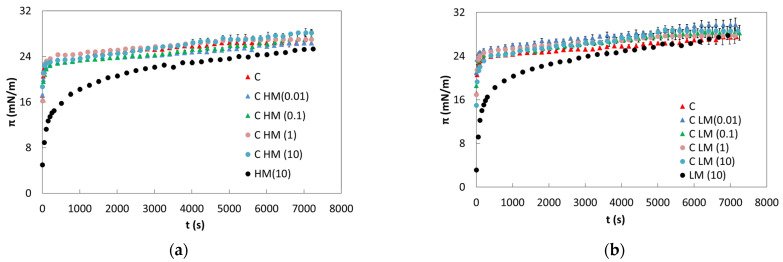
Surface pressure (π) of β-casein mixtures with HM pectin (**a**) and LM pectin (**b**) at several concentrations.

The surface activity of the protein–polysaccharide mixture was evaluated. The behavior of pure protein, and HM and LM pectin, was obtained and compared, and the trend showed agreement with the literature [23,24]. Specifically, β-casein exhibits saturation conditions at the A/W interface when present at 0.100 ± 0.002 g/L [23] with a surface tension value of 45.0 ± 0.2 mN/m. The saturated interface condition is reached when the molecules completely cover the free surface, and by measuring the surface tension values, it is verified that further additions of protein do not change the value. Concerning the pure investigated pectins, HM and LM both exhibit good surface activity and are capable of reducing the surface tension to an equilibrium value under saturation conditions (10 g/L) of 47.2 ± 0.4 and 44.5 ± 0.2 mN/m, respectively [24]. In Figure 1, the results of the surface pressure of the mixtures obtained with HM or LM pectin and 0.1 g/L of β-casein are shown. HM shows higher surface activity than LM, and this behavior is also found in the literature [22]. The difference in the surface pressure (π) seems to be consistent with the density of methoxyl groups, taking into account the molecular weight (MW) of the two pectins. The HM could have a higher number of potentially adsorbed segments.

It is possible to observe that π of the mixtures increases rapidly in the first few seconds with a trend very similar to those of the pure β-casein, suggesting that the diffusion process is β-casein controlled. The protein’s influence on interfacial behavior is also noticeable when examining the behavior of pure pectin, as the surface pressure of the mixed systems quickly reaches a plateau value.

As it is possible to observe, the effect obtained in the presence of the two pectins is very similar. In both cases, the surface pressure reaches a value very similar to the pure protein independently of the pectin type. In fact, addition at different levels of the polysaccharide does not produce a significant change in the surface pressure, indicating that the interface is probably saturated within a short time by β-casein, which quickly reaches the interface.

The trend observed at short observation times can be entirely attributed to the milk protein due to its flexibility and low molecular weight, in contrast to the larger size of the two pectins. Additionally, the pectins, being less flexible, encounter more difficulty in reaching and covering the interface [36].

From Equation (2), using surface tension data during time, it is possible to evaluate the diffusion rate of the investigated species. For all investigated samples, the diffusion rate is too fast to be evaluated with this method of analysis because the surface pressure becomes quickly higher than 10 mN/m [38]. This behavior is very common for protein species [6,8], leading to the conclusion that the diffusion mechanism of pectin is entirely overshadowed by β-casein. The diffusion rates of pure pectins are evaluable and the values are 0.787 ± 0.038 and 0.561 ± 0.024 mN·m^−1^·s^−0.5^ for LM and HM pectin, respectively. It can be observed that the rate of diffusion is higher for the LM than the HM pectin. The result could seem incoherent with the lower MW and the high methoxyl group number of the HM compared with the LM pectin, but it could be related to the special conformation of the adsorbing groups along the pectin backbone [22]. Observing the surface pressure values for both β-casein and the mixed systems, it is noticeable that they increase rapidly beyond approximately 15 mN/m. This threshold value suggests a transition from a monolayer, with a more expanded structure, to one in which the structure becomes more condensed, as reported in the literature [36].

Other phenomena are relevant after the interface coverage, such as absorption (penetration), unfolding, and eventually the rearrangement or aggregation [6,36,39]. By Equations (3) and (4), the adsorption and rearrangement rates were evaluated; the data are reported in Table 1.

The data reported in Table 1 show that the adsorption and rearrangement rate of β-casein alone is very high compared to the mixed systems, and the k_ads_ and k_rearr_ of the mixed systems decrease with the increase in the pectin concentration.

The adsorption is generally controlled by the unfolding of the protein or by the mechanism of polysaccharides’ location at the interface. Pectins are large molecules, and as a result, they migrate slowly to the interface. Due to the initially low amount of polymer adsorbed at the A/W interface, the formation of the gel–structure interface takes more time. This is confirmed by the trend of π, which reaches values comparable to pure proteins at around 5000 s [36]. The k_ads_ rates for the two pure pectins are similar while the k_rearr_ is lower for the HM pectin. This trend can be attributed to the lower number of methyl groups, as suggested by Baldino et al. [24]. The differences in surface characteristics of the pectins also contribute to facilitating rearrangement at the interface, particularly when the methoxyl groups are in low abundance.

When the mixed systems are analyzed, a competitive mechanism is evident between the protein and the HM or LM pectin. In fact, the adsorption and rearrangement rates decrease and this effect is probably related to the collapse of the protein monolayer and the subsequent formation of a multilayer between the molecules, as already observed in the literature [9]. Finally, it is observed that CLM mixed systems exhibit a higher absorption rate compared to the CHM samples, likely attributed to the lower number of methyl groups. This lower amount allows for increased interaction with the protein, providing greater availability for interfacial unfolding.

The rearrangement rate of pure β-casein and pectins decreases in the mixed systems. This could be linked to the interaction between protein and pectin, causing a slowdown in the unfolding and rearrangement kinetics typically observed in pure protein [36]. The increase in pectin concentration leads to a higher rate of rearrangement, in agreement with literature findings. Rafe and their coworkers [40] demonstrated a similar trend, where the presence of HM pectin in a β-lactoglobulin solution increased the rate of rearrangement.

### 2.2. Oscillating Dilatational Rheology

**Figure 2:**
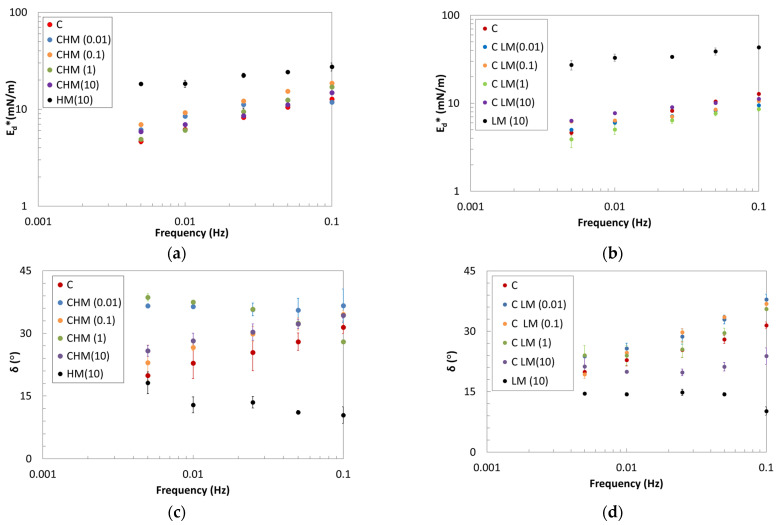
Frequency dependence of E_d_* moduli (**a**,**b**) and δ (**c**,**d**) of HM pectin–β-casein systems (**a**,**c**) and LM pectin–β-casein systems (**b**,**d**).

The mechanical resistance of the interfacial film of mixed systems was evaluated by dilatational kinematics, performing small-amplitude oscillations in the linear region. Frequency sweep tests were also performed, and the results are shown in Figure 2 for the pure biopolymers and the mixed systems. When comparing the two pure pectins, it is evident that the LM exhibits a higher complex modulus than the HM, with a similar degree of structuration that is also less frequency dependent, as seen in the δ trend (Figure 2c,d).

The two pectins can form resistant interfacial layers, solid-like and structured, as shown by the low values of the δ angle. On the contrary, the pure protein (C) forms a weaker interfacial layer, as seen by the lower E_d_* values and from the low frequency dependence. When surface pressure is considered, the mixed systems exhibit a behavior similar to that observed for the pure protein (see Section 2.1). On the other hand, with prolonged adsorption time, the monolayer formed by the protein in the initial stages could collapse, leading to cooperative adsorption with polysaccharide chains. Consequently, a thicker interface may be formed through association with β-casein in the adsorbed layer, as suggested by the rearrangement kinetic values [36].

The increase in E_d_* values for the mixed systems suggests the possibility of obtaining stronger interfaces. The magnitude of E_d_* is a consequence of the amount of adsorbed biopolymers at the interface. Therefore, the increment in the complex modulus, coupled with a decrease in the phase angle for the systems with protein and LM pectin, indicates robust macromolecular interactions among the adsorbed biopolymer segments.

Moreover, it can be observed that low concentrations of polysaccharides are sufficient to increase the moduli, owing to the larger size of polysaccharide segments compared to protein segments [36]. As the concentration of pectin increases, the moduli begin to decrease, probably because of the collapse of the monolayer [36], especially for HM pectin.

According to Equation (4), the strength and the structuration degree of the pure and mixed systems were evaluated. The *k* and *n* parameters of the critical gel model are reported in Table 2.

The parameters trend shows a very good mechanical resistance of the two pectins compared to the pure protein. It can be observed that the mixed systems have different behavior depending on the pectin type. HM pectin appears to enhance the consistency of the interface (higher k_d_) while reducing the structuring degree (higher n_d_), whereas LM pectin exhibits the opposite trend. The LM pectin seems able to give rise to complexes increasing the crosslinks, and therefore decreasing the n_d_ parameter, with a behavior that appears independent of the quantity of pectin in the mixture. Interfacial gel-structured systems, like CLM, exhibit enhanced resistance to dilatational deformations. The two polysaccharides adsorb at the A/W interface, contributing to a degree of structuration that may depend on differences in interfacial packing arising from methyl groups, molecular weight (MW), and the rearrangement of pectin in the presence of the protein. A similar trend was also observed in mixed systems of β-lactoglobulin with propylene glycol alginates and whey protein concentrate with hydroxypropylmethyl [22].

### 2.3. Oscillating Shear Rheology

The interfacial behavior was also analyzed in shear kinematics to study the long-term stability because, in general, proteins are not typically contributing to it [40]. The analysis was performed on the pure biopolymers and pectin/protein systems. Frequency sweep tests were performed at two different aging times, 0.5 h and 3.5 h, to understand the mixed systems behavior and the evolution of the interfaces.

As in dilatational analysis, the interface exhibits greater mechanical resistance in the presence of pure pectins compared to pure protein alone. Specifically, a solid-like behavior is observed in the presence of 1 and 10 g/L of LM pectin, whereas a liquid-like behavior is evident with pure β-casein. The systems with 0.1 and 0.01 g/L of LM show very weak behavior, and their interfaces remain unchanged with aging time, making analysis challenging. At concentrations of 1 and 10 g/L, the shear complex modulus and angle phase show an intermediate behavior between the values of pure β-casein and pure pectin, probably due to electrostatic interactions [40].

The films obtained by the mix of the biopolymers show, at a short time, a complex shear modulus intermediate between those of pure biopolymers at high pectin concentration. As for the dilatational kinematics, the angle phase is lower for systems with LM pectin, with respect to samples with HM, and it is possible to say that β-casein/LM pectin mixtures are mainly elastic (solid-like). The observed trend can be explained in terms of a synergistic interaction effect between biopolymers. The interaction among the adsorbed segments of the biopolymers is probably higher at a short aging time. In contrast, the β-casein/HM pectin system displays a weaker interface with an angle phase higher than that obtained with protein and LM pectin. Similar results were found for interfaces involving milk whey proteins/hydroxypropylmethyl cellulose, β-lactoglobulin, or milk whey proteins with high methoxyl pectin [22]. This behavior could be attributed to an antagonistic interaction effect between the protein and the hydrocolloid or the polysaccharide, which is particularly evident at low HM pectin concentrations.

**Figure 3:**
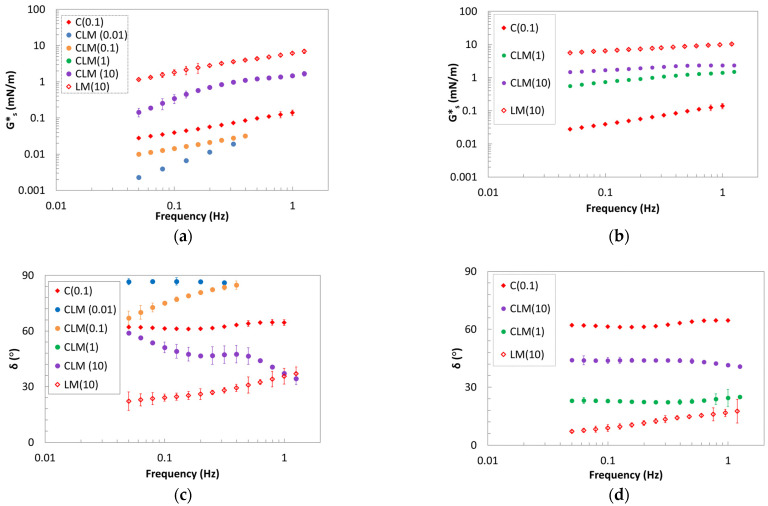
Frequency dependence of G_s_* moduli (**a**,**b**) and phase angle (**c**,**d**) of LM pectin–casein mixtures at 0.5 h (**a**,**c**) and 3.5 h (**b**,**d**) of aging.

**Figure 4:**
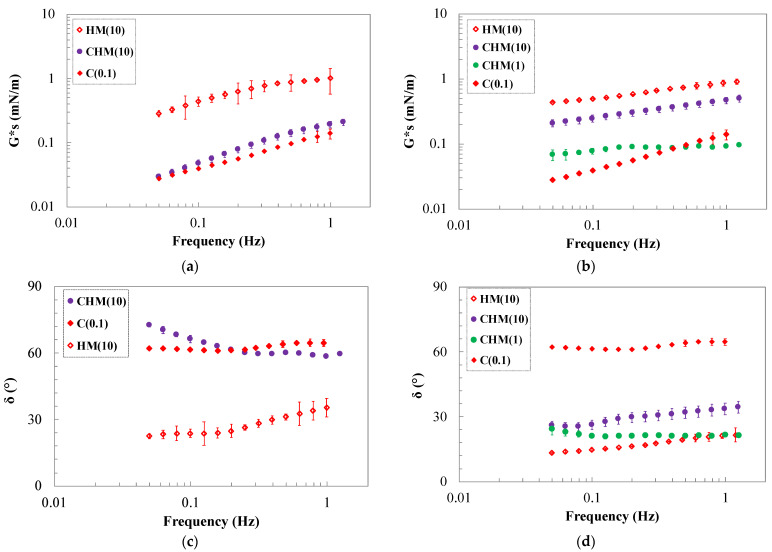
Frequency dependence of Gs* moduli (**a**,**b**) and phase angle (**c**,**d**) of HM pectin–casein mixtures at 0.5 h (**a**,**c**) and 3.5 h (**b**,**d**) of aging.

After aging for 3.5 h, as reported in Figure 3c,d, the CLM systems at higher concentrations exhibit an increase in their complex modulus, while the angle phase decreases. The gelation of the interface becomes evident due to the higher and less frequency-dependent G*_s_ and the lower δ (<45°) in all the mixed LM/β-casein systems. The interface after 3.5 h of evolution exhibits the typical behavior of a strong interface. The data presented in Figure 4 indicate that HM pectin promotes interactions, enhancing structurization, as confirmed by the phase angle at concentrations of 1 and 10 g/L. This yields a stronger film at the interface, characterized by a high complex modulus consistently showing values intermediate between those of the pure protein and the pure polysaccharide. Comparing the two mixtures, it becomes evident that in both cases, whether in the presence of HM or LM pectin, an evolving interface forms over time, gelling at concentrations of 1 and 10 g/L of pectin due to cooperative interactions. Furthermore, the system with casein is stabilized through the addition of polysaccharide.

Methoxyl groups, thanks to their strong hydrophobic nature, are able to penetrate with time and to rearrange at the air–water interface, giving rise to a strong gelled interface that can stabilize the system. Moreover, even if the β-casein has good interfacial activity and good viscoelastic interfacial film properties, at long-term adsorption, pectin with high DM could create resistant elastic surfaces according to other similar systems [22,40,41].

## 3. Conclusions

Protein–polysaccharide systems are increasingly employed in the food industry to achieve stable multiphasic systems over time. The physical stability of foams can be assessed through surface dilatational and shear rheology. Systems can be stabilized by increasing bulk viscosity or controlling interfacial structure. Consequently, we studied protein/polysaccharide mixtures using β-casein and HM and LM pectins.

The mixtures demonstrate high surface activity, with pectins noticeably influencing interfacial properties. The surface pressure analysis highlighted that the kinetics are strongly influenced by β-casein, which migrates to the interface and covers it very quickly. 

Dilatational data analysis shows that the behavior of the mixtures resembles that of protein alone more than that of pure pectins. However, interfacial moduli indicate improved viscoelastic behavior when pectins are added to the solution. Additionally, parameters such as k_d_ and n_d_ suggest stronger interfaces in the presence of HM pectins with a low structuring effect.

Shear kinematic analysis indicates that interfacial layers obtained in the presence of pectins are elastic and structured. This effect becomes more evident as the interface ages. Specifically, LM pectin creates stronger but less-structured interfacial gels compared with HM pectin. In every case, the addition of citrus pectin to the β-casein solution improves interfacial film properties, stabilizing it over time and promoting gelation at the interfaces.

## 4. Materials and Methods

### 4.1. Materials and Sample Preparation

The biopolymers used for the interfacial study are β-casein (Sigma-Aldrich, Saint Louis, MO, USA) and two commercial citrus pectins, kindly supplied by JRS Silvateam Ingredients srl (Cosenza, Italy). The β-casein sample was 98% pure and had a molecular weight of 24 kDa. The two commercial citrus pectins were HM (65.3% DM and 104 kDa MW) and LM (42.9% DM and 123 kDa MW). Two different pectins were chosen to observe the effect of different methoxylation degrees on protein. To guarantee complete solubilization, pectins were mixed for 12 h at room temperature in a citric buffer solution, at pH = 6 and ionic strength 0.1 M, using a heating magnetic stirrer (AREX, Velp scientific, Usmate (MB), Italy). For the buffer solution, Milli-Q water was used, and the surface tension was always verified and was 72.4 mN/m, the same as for pure water [42]. The β-casein solution was prepared by mixing in the citrus buffer for 1 h. The protein–polysaccharide mixtures were obtained by adding the protein to the pectin solutions, preparing as explained above, and then mixing the system for 1 h.

The β-casein solutions were prepared at 0.1 g/L concentration. For the protein–polysaccharide samples, constant protein concentrations of 0.1 g/L were used, while the pectin concentration ranged from 0.01 to 10 g/L. In addition, the two samples of pure pectin at the concentration of 10 g/L were analyzed. The protein concentration was chosen because it is the saturation condition of the pure β-casein, while the polysaccharide concentration range was chosen according to the literature [22,41] and to some commercial applications for which these quantities are interesting [36,43,44]. The value of the HM and LM pectins range where chosen according to the biopolymers quantity that can saturate the air–water interface on their own.

### 4.2. Static Measurements and Adsorption Kinetics

Surface tension measurements were performed using a pendant drop tensiometer (FTA200, First Ten Angstroms, Newark, CA, USA), equipped with fta32 v2.0 software, monitoring for 2 h a drop shape of the described samples at the interface with air, as in previous works [45,46]. From the surface tension measurements, the equilibrium surface tension value was obtained. In particular, the equilibrium condition was reached when the tension did not change by more than 0.5 mN/m in 30 min [24]. From the difference between the measured surface tension and the buffer surface tension, the surface pressure is obtained. With the same method, the equilibrium surface pressure was evaluated.

The transient interfacial tension profile allows the evaluation of kinetic parameters associated with the absorption phenomenon at the interface [8,45,47]. Diffusive mechanisms can be evaluated with the modified Ward–Tordai equation:

(1) $$\pi \left(t\right)=\gamma \left(t\right)-\gamma \left(t_{0}\right)=C_{0}KT\frac{D_{diff}t}{\Pi }$$

where γ(t) in the surface tension instantaneously measured and γ(t_0_) is the interfacial tension at t = 0, C_0_ is the bulk concentration in the aqueous phase, K is the Boltzmann constant, T is the absolute temperature, D_diff_ is the diffusion coefficient, Π the Pi Greco value, and t the adsorption time [48]. Plotting π(t) against t^1/2^, the trend will be linear if the diffusion is the mechanism that controls the adsorption process and the slope of this plot is the diffusion rate (k_diff_):

(2) $$k_{diff}=C_{0}KT{\left(\frac{D_{diff}}{\Pi }\right)}^{1/2}$$

**Figure 5:**
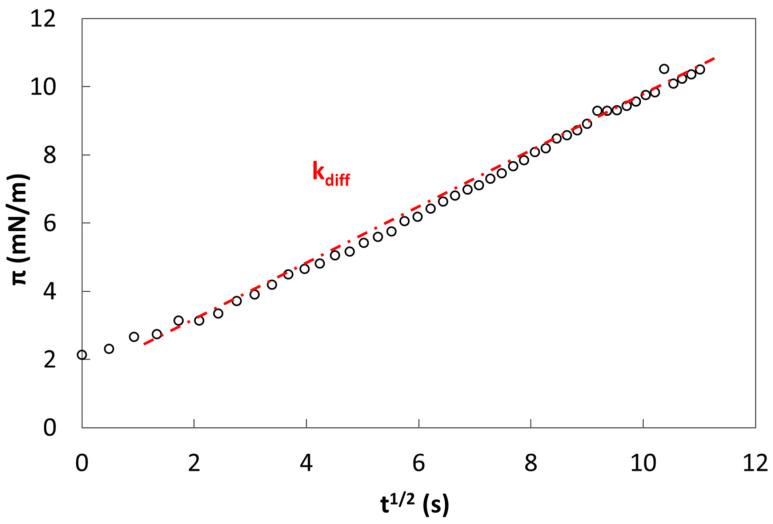
Diffusion rate determination k_diff_ by Ward–Tordai equation for pure LM pectin.

The graph in Figure 5 shows the diffusion rate determination interpreted with Equation (2), using the experimental surface tension values.

**Figure 6:**
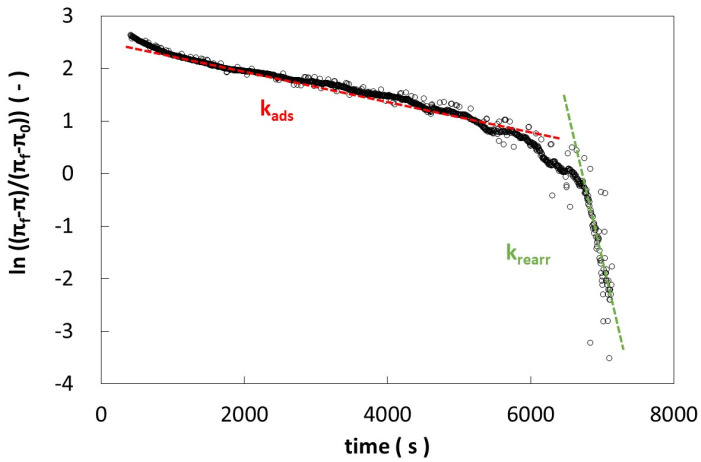
Example of adsorption (k_ads_) and rearrangement (k_rearr_) rates determination by Grahams and Philips equation for Ward–Tordai equation for pure LM pectin.

After diffusion, the absorption and rearrangement mechanisms were evaluated by the Graham and Phillips equation:

(3) $$ln\frac{\pi_{f}-\pi_{t}}{\pi_{f}-\pi_{0}}=-k_{i}t$$

where the surface pressures *π_f_*, *π_t_*, and *π*_0_ are evaluated at the end of the measure, at any given time, and at the beginning of the measurement, respectively [39], and k_i_ is the first-order rate constant. Based on the trend observed in the data plot, it is generally possible to discern two slopes: the first slope is associated with adsorption phenomena, denoted as k_ads_, while the second slope pertains to the rearrangement of molecules adsorbed within the interfacial layer, denoted as k_rearr_ [8,45,46]. The surface pressure values, after the diffusion period, are reported against time according to Equation (3) in Figure 6. As it is possible to observe, the curve in Figure 6 shows two slopes: the first slope represents the adsorption rate (k_i_ = k_ads_*)* and the second one represents the rearrangement (k_i_ = k_rearr_*)*.

### 4.3. Dilatational Rheological Analysis

In order to evaluate the mechanical strength of the surface layers under dilatational strain, measurements were performed in oscillatory mode using a pendant drop tensiometer. Small amplitude oscillations were applied through periodic expansions and compressions of a drop of aqueous solution suspended in air following the oscillating drop method [49,50]. Time sweep tests at different frequencies were performed to obtain the frequency sweep test within the 0.005–0.1 Hz range. Preliminary amplitude sweep tests were performed to identify the linear viscoelastic region (∆A/A = 10%) [49,50]. 

The rheological data were interpreted by using the bidimensional critical gel model, which describes the trend of E_d_* versus frequency, according to a power law trend in a log–log diagram [46,51]. According to this approach, we can use the following equation:

(4) $$E_{d}^{*}=k_{d}\cdot \omega^{n_{d}}$$

where *k_d_* is the interfacial dilatational gel strength and *n_d_* is its structuration degree. The data were fitted with the software TABLE CURVE 2D v 5.01 (Systat Software Inc., San Jose CA, USA).

### 4.4. Shear Rheological Analysis

Interfacial rheological measurements were performed also in shear kinematics, using an Interfacial Shear Rheometer (ISR400, KSV Instruments, Helsinki, Finland) equipped with a magnetic needle (length = 28.11 mm, weight = 0.0086 g) placed at the interface and moved by an imposed magnetic field, generated by two Helmotz coils [52]. The needle movement is instantly captured by an optical microscope, which measures the strain induced at the interface by the imposed magnetic field. The biopolymer solution was placed in a Petri plate and the magnetic needle was placed on the solution surface. According to our previous work [45], two different aging times, 0.5 and 3.5 h, were investigated. The aging times were evaluated by monitoring the time evolution of storage modulus, G′_s_, and loss modulus, G″_s_, by time sweep test. The first aging time (0.5 h) was chosen in the region where the interface was fresh and no changes were observable looking at the G′_s_ and G″_s_. After the first flat period, the moduli start to increase with time. The time evolution of storage and loss modulus by time sweep test was monitored until G′_s_ and G″_s_ showed a flat trend; then, the time sweep was stopped and a frequency sweep test was performed. The aging times were found to be similar for the two interfaces, CHM and CLM.

As in dilatational kinematic, the tests were performed in small-amplitude oscillation mode, in the linear region, preliminary evaluated by amplitude sweep tests. Dynamic time sweep tests at 0.1 Hz were performed to study the aging of the interface and to ensure the complete formation of the interfacial layers. Frequency sweep tests were performed in the 0.05–1.5 Hz frequency range. All tests were performed at room temperature (22 ± 1 °C).

## Figures and Tables

**Table 1:** Adsorption and rearrangement rate of protein–pectin mixed systems at A/W interface.

Pectin (g/L)	β-Casein (g/L)	k_ads_ × 10^4^ (s^−1^)		k_rearr_ × 10^4^ (s^−1^)	
		CHM	CLM	CHM	CLM
0	0.1	2.62 ± 0.01	2.62 ± 0.01	28.5 ± 0.6	28.5 ± 0.6
0.01	0.1	1.44 ± 0.03	2.12 ± 0.01	9.0 ± 0.2	9.3 ± 0.1
0.1	0.1	1.35 ± 0.01	1.56 ± 0.01	12.1 ± 0.1	12.1 ± 0.6
1	0.1	1.26 ± 0.02	1.34 ± 0.01	17.5 ± 0.3	12.5 ± 0.1
10	0.1	1.23 ± 0.01	1.49 ± 0.02	13.3 ± 0.3	12.1 ± 0.1
10	0	3.31 ± 0.09	3.04 ± 0.01	11.2 ± 0.05	19.43 ± 0.01

**Table 2:** Weak-gel parameters, k and n, of protein–pectin mixtures at A/W interface.

Pectin (g/L)	β-Casein (g/L)	k_d_ (mN/m·s)		n_d_ (-)	
		CHM	CLM	CHM	CLM
0	0.1	27 ± 1	27 ± 1	0.32 ± 0.01	0.32 ± 0.01
0.01	0.1	17 ± 2	15.3 ± 0.4	0.15 ± 0.04	0.21 ± 0.01
0.1	0.1	40 ± 1	15 ± 1	0.32 ± 0.01	0.19 ± 0.02
1	0.1	44 ± 1	15 ± 1	0.42 ± 0.01	0.24 ± 0.02
10	0.1	30 ± 2	16.9 ± 0.5	0.32 ± 0.02	0.17 ± 0.01
10	0	32 ± 3	59 ± 4	0.15 ± 0.02	0.14 ± 0.02

## Data Availability

The data used to support the findings of this study can be made available by the corresponding author upon request.

## References

[1] Bos M.A., Van Vliet T. (2001). Interfacial Rheological Properties of Adsorbed Protein Layers and Surfactants: A Review. Adv. Colloid Interface Sci..

[2] Dickinson E. (2011). Mixed Biopolymers at Interfaces: Competitive Adsorption and Multilayer Structures. Food Hydrocoll..

[3] McClements D.J. (2004). Food Emulsions: Principles, Practices, and Techniques.

[4] Ganzevles R.A., Cohen Stuart M.A., van Vliet T., de Jongh H.H.J. (2006). Use of Polysaccharides to Control Protein Adsorption to the Air-Water Interface. Food Hydrocoll..

[5] Dickinson E. (1999). Adsorbed Protein Layers at Fluid Interfaces: Interactions, Structure and Surface Rheology. Colloids Surf. B Biointerfaces.

[6] Perez A.A., Sánchez C.C., Patino J.M.R., Rubiolo A.C., Santiago L.G. (2010). Milk Whey Proteins and Xanthan Gum Interactions in Solution and at the Air–Water Interface: A Rheokinetic Study. Colloids Surf. B Biointerfaces.

[7] Bouyer E., Mekhloufi G., Rosilio V., Grossiord J.L., Agnely F. (2012). Proteins, Polysaccharides, and Their Complexes Used as Stabilizers for Emulsions: Alternatives to Synthetic Surfactants in the Pharmaceutical Field?. Int. J. Pharm..

[8] Camino N.A., Pérez O.E., Sanchez C.C., Rodriguez Patino J.M., Pilosof A.M.R. (2009). Hydroxypropylmethylcellulose Surface Activity at Equilibrium and Adsorption Dynamics at the Air-Water and Oil-Water Interfaces. Food Hydrocoll..

[9] Pérez O.E., Sánchez C.C., Pilosof A.M.R., Rodríguez Patino J.M. (2009). Kinetics of Adsorption of Whey Proteins and Hydroxypropyl-Methyl-Cellulose Mixtures at the Air–Water Interface. J. Colloid Interface Sci..

[10] Fischer P. (2013). Rheology of Interfacial Protein-Polysaccharide Composites. Eur. Phys. J. Spec. Top..

[11] Rodríguez Patino J.M., Pilosof A.M.R. (2011). Protein–Polysaccharide Interactions at Fluid Interfaces. Food Hydrocoll..

[12] Liang L., Luo Y. (2020). Casein and Pectin: Structures, Interactions, and Applications. Trends Food Sci. Technol..

[13] Cheng Y., Liu D., Zeng M., Chen J., Mei X., Cao X., Liu J. (2022). Milk β-Casein as Delivery Systems for Luteolin: Multi-Spectroscopic, Computer Simulations, and Biological Studies. J. Food Biochem..

[14] Singh H. (2011). Aspects of Milk-Protein-Stabilised Emulsions. Food Hydrocoll..

[15] Bantchev G.B., Schwartz D.K. (2003). Surface Shear Rheology of β-Casein Layers at the Air/Solution Interface: Formation of a Two-Dimensional Physical Gel. Langmuir.

[16] de Kruif C.G., Huppertz T., Urban V.S., Petukhov A.V. (2012). Casein Micelles and Their Internal Structure. Adv. Colloid Interface Sci..

[17] Głąb T.K., Boratyński J. (2017). Potential of Casein as a Carrier for Biologically Active Agents. Top. Curr. Chem..

[18] Dauphas S., Mouhous-Riou N., Metro B., MacKie A.R., Wilde P.J., Anton M., Riaublanc A. (2005). The Supramolecular Organisation of β-Casein: Effect on Interfacial Properties. Food Hydrocoll..

[19] Bhat M.Y., Dar T.A., Singh L.R., Dar T.A. (2016). Casein Proteins: Structural and Functional Aspects. Milk Proteins.

[20] Eskin N.A.M., Goff H.D., Eskin N.A.M., Shahidi F. (2013). Chapter 4—Milk. Biochemistry of Foods.

[21] Beverung C.J., Radke C.J., Blanch H.W. (1999). Protein Adsorption at the Oil/Water Interface: Characterization of Adsorption Kinetics by Dynamic Interfacial Tension Measurements. Biophys. Chem..

[22] Perez A.A., Sánchez C.C., Rodríguez Patino J.M., Rubiolo A.C., Santiago L.G. (2011). Surface Adsorption Behaviour of Milk Whey Protein and Pectin Mixtures under Conditions of Air-Water Interface Saturation. Colloids Surf. B Biointerfaces.

[23] Baldino N., Mileti O., Lupi F., Gabriele D., Ahmed J., Basu S. (2023). Chapter 1—Interfacial Rheology of Food: Protein as a Model Food. Advances in Food Rheology and Its Applications.

[24] Baldino N., Mileti O., Lupi F.R., Gabriele D. (2018). Rheological Surface Properties of Commercial Citrus Pectins at Different PH and Concentration. LWT.

[25] Thakur B.R., Singh R.K., Handa A.K., Rao M.A. (1997). Chemistry and Uses of Pectin—A Review. Crit. Rev. Food Sci. Nutr..

[26] Alba K., Kontogiorgos V. (2017). Pectin at the Oil-Water Interface: Relationship of Molecular Composition and Structure to Functionality. Food Hydrocoll..

[27] Maxwell E.G., Belshaw N.J., Waldron K.W., Morris V.J. (2012). Pectin—An Emerging New Bioactive Food Polysaccharide. Trends Food Sci. Technol..

[28] Willats W.G.T., Knox J.P., Mikkelsen J.D. (2006). Pectin: New Insights into an Old Polymer Are Starting to Gel. Trends Food Sci. Technol..

[29] Ngouémazong E., Christiaens S., Shpigelman A., van Loey A., Hendrickx M. (2015). The Emulsifying and Emulsion-Stabilizing Properties of Pectin: A Review. Compr. Rev. Food Sci. Food Saf..

[30] Siew C.K., Williams P.A. (2008). Role of Protein and Ferulic Acid in the Emulsification Properties of Sugar Beet Pectin. J. Agric. Food Chem..

[31] Gawkowska D., Cybulska J., Zdunek A. (2018). Structure-Related Gelling of Pectins and Linking with Other Natural Compounds: A Review. Polymers.

[32] Lupi F.R., Gabriele D., Seta L., Baldino N., de Cindio B., Marino R. (2015). Rheological Investigation of Pectin-Based Emulsion Gels for Pharmaceutical and Cosmetic Uses. Rheol. Acta.

[33] Fissore E.N., Rojas A.M., Gerschenson L.N., Williams P.A. (2013). Butternut and Beetroot Pectins: Characterization and Functional Properties. Food Hydrocoll..

[34] Funami T., Zhang G., Hiroe M., Noda S., Nakauma M., Asai I., Cowman M.K., Al-Assaf S., Phillips G.O. (2007). Effects of the Proteinaceous Moiety on the Emulsifying Properties of Sugar Beet Pectin. Food Hydrocoll..

[35] Yapo B.M., Robert C., Etienne I., Wathelet B., Paquot M. (2007). Effect of Extraction Conditions on the Yield, Purity and Surface Properties of Sugar Beet Pulp Pectin Extracts. Food Chem..

[36] Baeza R., Pilosof A.M.R., Sanchez C.C., Rodríguez Patino J.M. (2006). Adsorption and Rheological Properties of Biopolymers at the Air-Water Interface. AIChE J..

[37] Ganzevles R.A., Fokkink R., van Vliet T., Cohen Stuart M.A., de Jongh H.H.J. (2008). Structure of Mixed β-Lactoglobulin/Pectin Adsorbed Layers at Air/Water Interfaces; a Spectroscopy Study. J. Colloid Interface Sci..

[38] Seta L., Baldino N., Gabriele D., Lupi F.R., de Cindio B. (2014). Rheology and Adsorption Behaviour of β-Casein and β-Lactoglobulin Mixed Layers at the Sunflower Oil/Water Interface. Colloids Surf. A Physicochem. Eng. Asp..

[39] Graham D.E., Phillips M.C. (1979). Proteins at Liquid Interfaces II: Adsorption Isotherms. J. Colloid Interface Sci..

[40] Rafe A., Selahbarzin S., Kulozik U., Hesarinejad M.A. (2022). Dilatational Rheology-Property Relationships of β-Lactoglobulin /High Methoxyl Pectin Mixtures in Aqueous Foams. Food Hydrocoll..

[41] Arboleya J.-C., Wilde P.J. (2005). Competitive Adsorption of Proteins with Methylcellulose and Hydroxypropyl Methylcellulose. Food Hydrocoll..

[42] Lin S.-Y., Wang W.-J., Lin L.-W., Chen L.-J. (1996). Systematic Effects of Bubble Volume on the Surface Tension Measured by Pendant Bubble Profiles. Colloids Surf. A Physicochem. Eng. Asp..

[43] Saha D., Bhattacharya S. (2010). Hydrocolloids as Thickening and Gelling Agents in Food: A Critical Review. J. Food Sci. Technol..

[44] Arboleya J.C., García-Quiroga M., Lasa D., Oliva O., Luis-Aduriz A. (2014). Effect of Highly Aerated Food on Expected Satiety. Int. J. Gastron. Food Sci..

[45] Mileti O., Baldino N., Carmona J.A., Lupi F.R., Muñoz J., Gabriele D. (2022). Shear and Dilatational Rheological Properties of Vegetable Proteins at the Air/Water Interface. Food Hydrocoll..

[46] Seta L., Baldino N., Gabriele D., Lupi F.R., De Cindio B. (2012). The Effect of Surfactant Type on the Rheology of Ovalbumin Layers at the Air/Water and Oil/Water Interfaces. Food Hydrocoll..

[47] Mileti O., Baldino N., Lupi F.R., Gabriele D. (2023). Interfacial Behavior of Vegetable Protein Isolates at Sunflower Oil/Water Interface. Colloids Surf. B Biointerfaces.

[48] Ward A.F.H., Tordai L. (1946). Time-Dependence of Boundary Tensions of Solutions I. The Role of Diffusion in Time-Effects. J. Chem. Phys..

[49] Derkach S.R., Krägel J., Miller R. (2009). Methods of Measuring Rheological Properties of Interfacial Layers (Experimental Methods of 2D Rheology). Colloid J..

[50] Ravera F., Loglio G., Kovalchuk V.I. (2010). Interfacial Dilational Rheology by Oscillating Bubble/Drop Methods. Curr. Opin. Colloid Interface Sci..

[51] Dicharry C., Arla D., Sinquin A., Graciaa A., Bouriat P. (2006). Stability of Water/Crude Oil Emulsions Based on Interfacial Dilatational Rheology. J. Colloid Interface Sci..

[52] Brooks C.F., Fuller G.G., Frank C.W., Robertson C.R. (1999). Interfacial Stress Rheometer to Study Rheological Transitions in Monolayers at the Air-Water Interface. Langmuir.

